# Traumatic Brain Injury in Mice Induces Acute Bacterial Dysbiosis Within the Fecal Microbiome

**DOI:** 10.3389/fimmu.2018.02757

**Published:** 2018-11-27

**Authors:** Todd J. Treangen, Justin Wagner, Mark P. Burns, Sonia Villapol

**Affiliations:** ^1^Department of Computer Science, Rice University, Houston, TX, United States; ^2^Center for Bioinformatics and Computational Biology, University of Maryland, College Park, MD, United States; ^3^Department of Neuroscience, Georgetown University, Washington, DC, United States; ^4^Center for Neuroregeneration, Houston Methodist Research Institute, Houston, TX, United States

**Keywords:** microbiome, gut-brain axis, Lactobacillus, brain damage, gut microbes, traumatic brain injury, bacterial dysbiosis, controlled cortical impact injury

## Abstract

The secondary injury cascade that is activated following traumatic brain injury (TBI) induces responses from multiple physiological systems, including the immune system. These responses are not limited to the area of brain injury; they can also alter peripheral organs such as the intestinal tract. Gut microbiota play a role in the regulation of immune cell populations and microglia activation, and microbiome dysbiosis is implicated in immune dysregulation and behavioral abnormalities. However, changes to the gut microbiome induced after acute TBI remains largely unexplored. In this study, we have investigated the impact of TBI on bacterial dysbiosis. To test the hypothesis that TBI results in changes in microbiome composition, we performed controlled cortical impact (CCI) or sham injury in male 9-weeks old C57BL/6J mice. Fresh stool pellets were collected at baseline and at 24 h post-CCI. 16S rRNA based microbiome analysis was performed to identify differential abundance in bacteria at the genus and species level. In all baseline vs. 24 h post-CCI samples, we evaluated species-level differential abundances via clustered and annotated operational taxonomic units (OTU). At a high-level view, we observed significant changes in two genera after TBI, *Marvinbryantia*, and *Clostridiales*. At the species-level, we found significant decreases in three species (*Lactobacillus gasseri, Ruminococcus flavefaciens*, and *Eubacterium ventriosum)*, and significant increases in two additional species (*Eubacterium sulci*, and *Marvinbryantia formatexigens)*. These results pinpoint critical changes in the genus-level and species-level microbiome composition in injured mice compared to baseline; highlighting a previously unreported acute dysbiosis in the microbiome after TBI.

## Introduction

Traumatic brain injury (TBI) is a major cause of death and disability that represents one of the most prevalent injury types sustained by the worldwide population (1). It is known to cause a massive neuronal loss and oxidative stress in the cortical region around the site of the impact formed (2, 3). Brain injuries also cause imbalances and gastrointestinal dysfunction (4). Specifically, gut barrier dysfunction results from high plasma levels of endotoxins and increased intestinal permeability following TBI (5), and evidence also points to an effect of commensal gut microbiota on the brain, now often referred to as the microbiome-gut-brain axis.

The bidirectional influence of the gut-brain axis that modulates the neuroinflammatory process occurring at the time of the TBI and over hours, days, or weeks that follow. The gut microbiota can also influence brain function and behavior through the peripheral and central immune system, affecting to the circulating cytokines, which can act on corresponding receptors in neurons, glial cells, and endothelial cells to induce behavioral changes (6–8). As an example, the gut microbiome has emerged as a potent regulator of the immune system; inflammatory changes that occur after brain trauma are immediate and illustrate how cortical injury can lead to inflammatory consequences in the lining of the gastrointestinal mucosa (9). Gut microbiota likely plays a role in regulating intestinal barrier function that prevents the penetration of pathogenic compounds, and intestinal barrier dysfunction has been associated as a consequence of TBI (10). Healthy gut microbiota is critical for preventing bacterial translocation. Regulating diversity and balance in the healthy gut is still an active area of research, pioneered by the Human Microbiome Project (11) and MetaHIT (12, 13) projects that surveyed an extensive collection of healthy and unhealthy adults to get a better understanding of what it means to have a “healthy” gut microbiome (14).

Recently, the gastrointestinal system has been identified as being impacted by brain injury. Evidence supporting a bi-directional communication between the gut and brain includes changes to the mouse microbiome in animal models of brain ischemia (15) and spinal cord injury (14) and how intestinal dysbiosis alters immune homeostasis and injury recovery (6, 16). There is no data on the gut microbiome in TBI, however moderate to severe brain trauma in humans reduces gastric emptying (17) and intestinal contractility (18). Experimental TBI in mice caused a breakdown in the gut barrier with increased intestinal permeability, resulting in high levels of endotoxins (5, 19).

Changes to the gut microbiome represent a therapeutic avenue for the treatment of TBI due to the communication between injured brain and disruptions of the gut microbiome and its relation on the neuropathology of injury. In this study, we have analyzed microbial changes that occur 24 h after TBI in mice. Our results demonstrate that CCI causes a rapid shift in relative abundance of five species, including changes in the diversity of the psychoactive *Lactobacillaceae* family and protective *Lachnospiraceae* family, both commonly found in the human gut microbiome.

## Methods

### Mice and controlled cortical impact injury

9-weeks-old male C57BL/6J mice were housed in a room maintained at a condition with 12 h light dark cycle, two mice per cage, eight mice per group. Daily fecal samples were pooled and redistributed amongst all of the experimental cages. Controlled Cortical Impact (CCI) injury was conducted as previously described (20). Sham mice received all procedures except contusion. Feces samples were obtained from each mouse temporarily housed individually in a sterile cage without bedding and collected in sterile tubes 1 day before and 1 day after CCI injury. Mice were euthanized with CO_2_ following the Institutional Animal Care and Use Committee (IACUC) a standard procedure for euthanasia. All animal experiments were performed in accordance with approved protocol # 2017-0077 from Georgetown University Institutional Animal Care and Use Committee and comply with the approved institutional guidelines and regulations.

### Fecal DNA extraction

Genomic bacterial DNA was isolated from frozen stool samples using the QIAamp Fast DNA Stool Mini Kit (Qiagen). Snap-frozen fecal samples stored at −80°C were added to sterile tubes and treated as described in the manufacturer's instructions. The tubes containing the pretreated samples were placed into a homogenizer and disrupted. The concentration of the extracted genomic DNA was qualified with Nanodrop 2000 UV spectrophotometer (Thermo Scientific).

### Sequencing of 16S rRNA V3–V4 regions

The gene-specific sequences used in this protocol target the 16S V3 and V4 region. Sequencing libraries of the V3–V4 region were prepared according to the Illumina MiSeq system instructions. 16S bacterial rRNA gene were amplified using polymerase chain reaction (PCR) amplification with V3 and V4 region primers: (forward: 5' TCGTCGGCAGCGTCAGATGTGTATAAGAGACAGCCTACGGGNGGCWGCAG; and reverse:5'GTCTCGTGGGCTCGGAGATGTGTATAAGAGACAGGACTACHVGGGTATCTAATCC) for the first PCR and Nextera XT. Amplicons were generated using primers corresponding to the hypervariable regions, and the PCR products were purified. A library was created by targeting the 16S V3 and V4 regions. Sequencing was performed via Illumina MiSeq platform using a 300 bp paired-end libraries.

### Operational taxonomic units (OTU) and statistical analyses

The sequences generated from PCR-amplified 16S rRNA genes were quality inspected and filtered using Trimmomatic. We clustered the reads using DNAclust (21) into OTUs, normalized using Cumulative Sum Scaling, and assessed differential abundance with metagenomeSeq. QIIME2 was used to perform alpha and beta diversity analyses (qiime diversity core-metrics) and longitudinal (qiime longitudinal pairwise-differences) and volatility analyses (qiime longitudinal volatility). Log-fold change analysis and feature inspection were performed with MetaViz platform (22) and, interactive relative abundance plots were generated using Krona (23) and QIIME2 (24). Statistically significant differences in species-level relative abundance between paired samples were identified both using the non-parametric Wilcoxon ranked sum test and metagenomeSeq (25).

## Results

### Genus-level changes after traumatic brain injury

**Figure 1:**
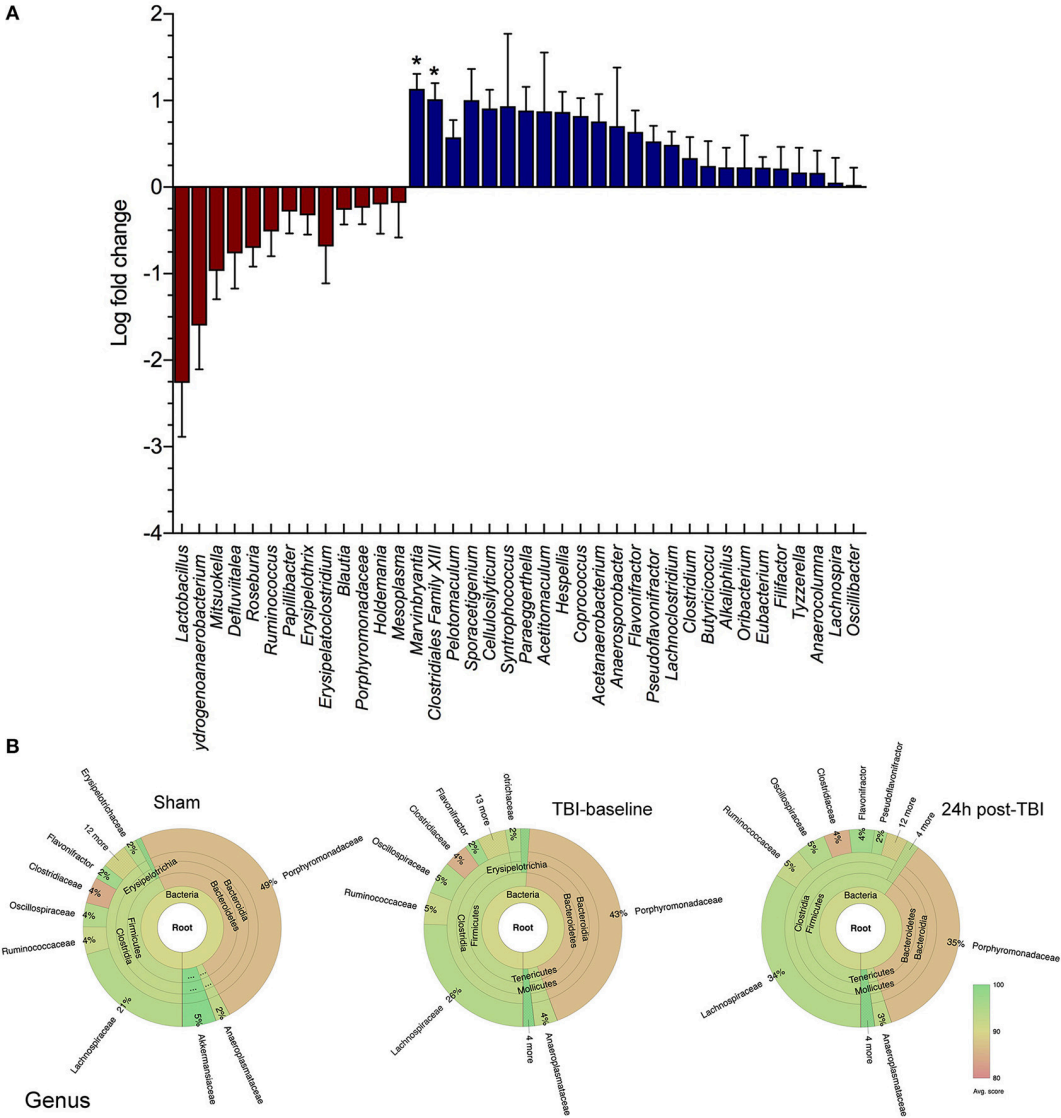
Family and Genus level changes before and after brain injury. **(A)** Box and whiskers plot shows the microbial community analysis using short-read (Illumina) sequencing comparing TBI and Sham animals at baseline levels and 24 h after brain injury (*n* = 8/group). The genus Lactobacillus relative abundance is noticeably decreased, by up to >2 log fold change (*p* > 0.05). The *Marvinbryantia* (*p* = 0.02) and *Clostridiales* genera (^*^p < 0.05) both significantly increase after TBI. **(B)** Krona chart highlights the genus differential abundance in Sham mice, TBI-baseline and 24 h post-TBI mice. TBI-baseline and Sham exhibit similar compositions, while 24 h post-TBI Krona plot highlights a shift in the community composition.

We have characterized the mouse gut microbiome using 16S rRNA analysis of multiple stool samples following experimental TBI. We found numerous bacterial species with significant log fold decreases in abundance 24 h post-TBI compared to either sham mice (baseline and 24 h) or their baseline (Figure 1A). Specifically, the *Lactobacillus* genus relative abundance noticeably decreased, by up to a 2 log fold change, but this change was not statistically significant (*p* > 0.05). However, *Marvinbryantia* (*p* < 0.05) and *Clostridiales* genera (*p* < 0.05) both significantly increase after TBI (Figure 1A). The Krona chart highlights the genus differential abundance in sham mice, TBI-baseline and 24 h post-TBI mice. TBI-baseline and sham exhibit similar compositions, while 24 h post-TBI Krona plot highlights a subtle shift in the community composition (Figure 1B).

### Species-level changes after traumatic brain injury

**Figure 2:**
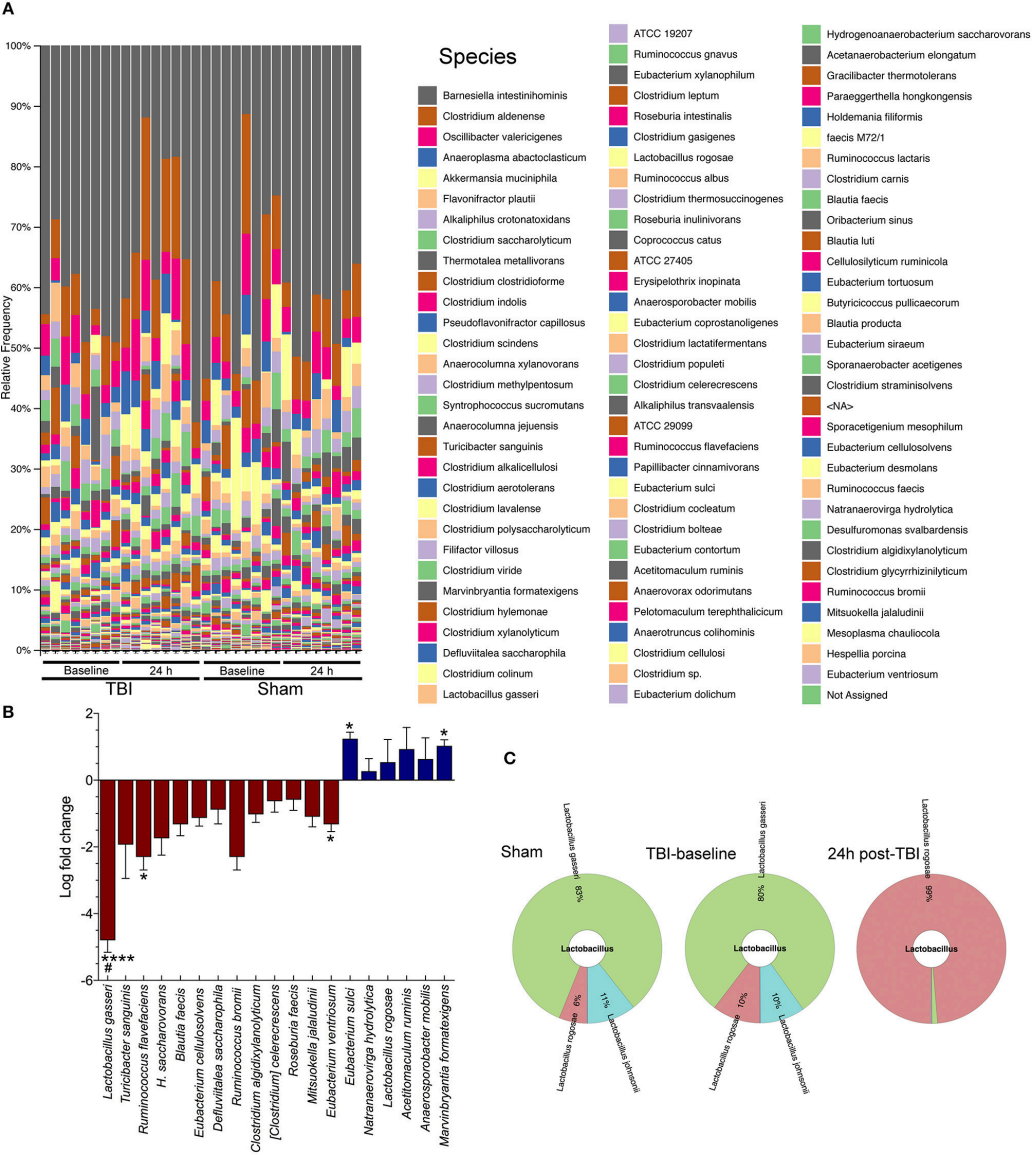
Species-level changes after acute brain injury. **(A)** The legend indicates each detected bacterial species, ordered from highest relative abundance to lowest relative abundance (colors are repeated). Gray colored bars at the top of each column in the bar plot represents the highest relative abundance of any species: *Barnesiella* intestinihominis. **(B)** TBI causes a significant decrease in *Lactobacilus gasseri* (^****^p < 0.0001), *Ruminococcus flavefaciens* (^*^p < 0.05), and *Eubacterium ventriosum* (^*^p < 0.05) compared to baseline levels; and a significant increase in *Eubacterium sulci* (^*^p < 0.05), and *Marvinbryantia formatexigens* (^*^p < 0.05). TBI also caused a decrease in *L. gasseri* (#*p* < 0.05) with the sham group (baseline and 24 h), (*n* = 8/group). **(C)** Krona chart highlights the relative differential abundance within the Lactobacillus family after injury, showing a near complete loss of *L. gasseri, johnsonii*, and *taiwainensis* bacteria, leaving *L. rogosae* as the lone representative in that genus after TBI.

The QIIME species barplot (Figure 2A) shows the overall species-level relative abundance across both TBI and sham mice, at baseline and 24 h, with little to no noticeable patterns in differential abundances across samples. However, when we compared TBI group basal samples to TBI-24 h, we observed a significant change in four bacterial species (Figure 2B). Specifically, TBI caused a significant decrease in *Lactobacillus gasseri* (^****^p < 0.0001), *Ruminococcus flavefaciens* (^*^p < 0.05), and *Eubacterium ventriosum* (^*^p < 0.05) compared to baseline levels; and a significant increase in *Eubacterium sulci* (^*^p < 0.05), and *Marvinbryantia formatexigens* (^*^p < 0.05). TBI also caused a decrease in *L. gasseri* (^#^*p* < 0.05) with significant differences observed in the sham group, indicating a possible shared stress response. *Lactobacillus gasseri* after TBI decreased by more than a 4-fold log change compared to basal animals (metagenomeSeq) (Figure 2B). The Krona chart (Figure 2C) highlights the relative differential abundance within the Lactobacillus family after injury, showing a near complete loss of *Lactobacillus gasseri, johnsonii, and taiwainensis* bacteria, leaving *L. rogosae* as the lone representative in that genus after TBI. Specifically, *L. gasseri* is the predominant member of the *Lactobacillus* genus in sham and TBI-baseline mice, making up over 80% of the *Lactobacillus* population (Figure 2C).

## Discussion

In this study, we describe acute changes in the gut microbiome after brain damage in mice. Specifically, our findings suggest that specific commensal microbiota might play a role in the recovery from brain injury. Although, a link between TBI and intestinal dysfunction was previously demonstrated (5, 19), the focus of attention has shifted to gut microbiota as a critical factor in the inflammatory, immunological, or anxiety-related response and post-injury depression (26, 27).TBI causes a rapid shift in microbiota diversity within 24 h of brain injury, including a dramatic change in the diversity of the psychoactive Lactobacillus family (Figure 2). We found significant increases and decreases after TBI. Most of the identified differentially abundant species, including *L. gasseri, M. formatexigens*, and *E. ventriosum*, are known to inhabit the human gut microbiome (28), indicating translational potential. If follow up studies prove fruitful; these bacteria could potentially be translated to human TBI patients for improved neuroinflammatory and neurological recovery.

We also observed significant changes in multiple bacterial species. We found *L. gasseri* after TBI to decrease by a more than 4-fold log change compared to basal animals. Probiotic *Lactobacillus* strains have been associated with the attenuation of anxiety deficits (29, 30). Also, previous studies have demonstrated that *Lactobacillus* and *Bifidobacterium* species may prevent chronic psychological stress, reduce apoptosis in several brain regions, and improve learning and memory in mice (29, 31). Curiously, *L. gasseri* was also decreased in sham mice after 24 h. This decrease could be a consequence of stress as it was previously described (32), due to the handling and anesthesia of the sham mice that cause stress. *Ruminococcus flavieciens* was also found to be decreased after brain injury. This bacterium is most commonly found in the rumen of wild and domesticated animals and has been previously associated with stress response (33). A decrease of *Ruminococcus* spp was previously documented in amyotrophic lateral sclerosis patients with signs of intestinal inflammation (34). Also, patients with Crohn's disease have been found to have lower levels of *Ruminococcus albus* than healthy individuals (35). We also found an increase of *E. ventriosum* in the injured mice. This bacterium was previously found to be increased in obese adults (36, 37) suggesting that gut microbiota composition is related to obesity. We did not find a correlation between the increased relative abundance of *E. ventriosum and weight*. Finally, our results also highlight an increase in the *M. formatexigens* species in mice after TBI. *M. formatexigens* consumes oligosaccharides, does not impact the redox state of the gut, and boosts the yield of succinate (38). Interestingly, succinate receptors have been found in the gut epithelium, representing a potential therapeutic target for the succinate toxicity (39). These receptors may mediate the local stress responses, including cerebral ischemia, hypoxia or TBI. A recent study using a rat model of TBI found phyla-level changes in alpha-diversity at 2 h post-injury (40). The small number of sham animals (4 per group) requiring follow-up studies to confirm these complementary findings.

Probiotics may confer a health benefit to the brain function and involved in the maintenance of the diversity of gut microbiota (41, 42). Our findings raise the intriguing possibility that probiotics may confer a health benefit to the brain following TBI. Data from animal models of stroke and spinal cord injury support the hypothesis that injury to the CNS causes a downstream effect on the gut microbiome (15) and treatments targeting biodiversity in the gut can impact CNS injury outcome (6, 16). Though not yet explored for TBI, probiotic treatment has been shown to affect the immune response by altering the Th1/Th2 imbalance (43).

In summary, the role of microbes in gut-brain cross-talk and the pathology of brain trauma represents a therapeutic target for recovery from TBI. Based on these preliminary findings, we hypothesize that repairing gut dysbiosis caused by TBI using probiotic treatments might serve as a new tool for reducing brain inflammation and improving anxiety and depression phenotypes after TBI. Future follow-up studies will be required to expand on research avenues indicated by our initial findings.

## Ethics statement

All procedures were performed in accordance with protocols approved by the Georgetown University Animal Care and Use Committee.

## Author contributions

TT, JW, MB, and SV conducted all the experiments. MB and SV performed the animal surgery, and SV performed bacterial DNA purification. TT and JW performed the bioinformatic and statistical analyses. TT and SV designed the study, analyzed the data, interpreted the results, and wrote the manuscript. All authors contributed to the editing of the manuscript. All the authors read and approved the final version before submission.

### Conflict of interest statement

The authors declare that the research was conducted in the absence of any commercial or financial relationships that could be construed as a potential conflict of interest.
